# Clinical Lecture*Specially reported for the Boston Medical and Surgical Journal.

**Published:** 1883-03

**Authors:** David W. Cheever

**Affiliations:** Professor of Surgery, Harvard University


CLINICAL LECTURE*. By DAVID W. CHEEVER, M. D., Professor of Surgery, Harvard University.

### I. CELLULAR ABSCESS.

*Gentlemen :*—The first case which I bring before you to-day is that of this little girl, who came here a week ago with a hard bunch upon her left wrist. This bunch has now softened. It gives rise to great apparent deformity and protrusion of the ulna, which appearance, however, is deceptive. The wrist-joint moves freely in all directions, without pain. There is a sac containing pus, which I proceed to incise. If the pus is due to caries, the disease at all events does not affect the joint, and it is doubtless merely an inflammation of the cellular tissue.

### II. PUNCTURED WOUND OF HAND; LIGATURE OF THE BRACHIAL ARTERY.

This little boy, about six years of age, nearly three weeks ago drove a penknife into his hand, in the neighborhood of the

---

* Specially reported for the Boston Medical and Surgical Journal.

first carpo-metacarpal articulation. The immediate hæmorrhage was slight. One week after the injury, however, profuse secondary hæmorrhage occurred. The physicians in attendance prolonged the original incision from the *tabatiere* toward the seat of the radial artery in the wrist, and made an attempt to secure the bleeding vessel, but without success. A firm compress was then applied to the hand, and the boy was sent from his home, two hundred miles distant, to Boston, where he arrived exhausted and pale from the amount of blood that had been lost. On taking off the twist from the hand I found a foul suppurating cavity at the site of the incision; also a superficial slough on the ulnar side of the hand, due to pressure of the bandage. No blood appeared from the vessel, but there was reason, in view of the bad condition of the parts, to suppose that hæmorrhage would recur. I I therefore ligatured the brachial artery. The operation was performed under the spray, and Lister dressings were applied. This was eleven days ago. While there has not been absolutely first intention, the wound has practically healed, as you see, the granulations having come up to the level of the skin. The ligature, which was of catgut, never came away, and there is reason to believe that it has been absorbed. There is no further danger, now, of secondary hæmorrhage, and the child can go to his home in a few days.

There are two points worthy of attention in this case. First, the safety of tying the brachial, so far as concerns danger of mortification of the hand, and its advantages as serving to secure the interosseous arteries. By applying the ligature to this vessel, we also avoid the neighborhood of the sloughing tissues about the wound. Secondly, the prompt establishment of the collateral circulation. On the eighth day after the operation I detected pulsation in the ulnar artery, and on the ninth it was observed in the radial. It is now perfectly restored in both. In this case the nails have retained their bright pink color throughout, and the hand has been warm.

### III. ULCER OF THE UPPER LIP.

This man as you will observe, has upon his upper lip an ulcer, with ragged, hard edges. It has been there for six or seven months. He has also had for the past three months venereal sores upon his privates, which have not healed yet. These sores, however, seem to be antedated by that upon the lip. The question in this case is whether the man has a primary specific sore upon the lip, or whether the ulcer is a degenerating epithelial growth. The patient himself ascribes the sore to irritation from a pipe, aggravated by neglect. I can detect no enlarged glands in the neck, and there appears to be no soreness on either side.

It is important to decide on the nature of this sore within a few weeks for the sake of the treatment. The man says he has taken no medicine. We will therefore put him at once upon a course of mercurials, and follow it up vigorously for three or four weeks, either alone or in connection with the iodide. If by that time the sore has not begun to heal, it should be excised.

### IV. ACUTE SYNOVITIS OF THE WRIST.

This lad is in the employ of a tailor. He tells me that he is not called upon to do heavy lifting or other work which would be likely to strain his wrist. He had previously been in good health, when suddenly, six days ago, he was taken with pain in the wrist, without having had any injury. Stiffness, swelling, and deformity came on at once, preventing the use of the hand. The hand appears pushed over to the radial side. The swelling is confined to the ulner side, and there is great tenderness through the ulnar articulation. He tells me that this is the only point affected, and that he never has had rheumatism. The localization of the trouble makes it look like acute synovitis, which, in the absence of any strain or injury, may be of rheumatic origin. As to the treatment, we will begin with the theory of rheumatism. The hand should be placed on a splint; we will give him colchicum internally, and locally lotions of laudanum and camphor, to be followed later by iodine. If the disease is rheumatism it will probably be relieved by this treatment. If synovitis it will assume an indolent type, and continue a long time.

### V. COMPLETE CONGENITAL EXTROVERSION OF THE BLADDER.

While the next patient is being etherized I will say a few words above the case, and will then demonstrate the pathological condition, and afterwards perform the operation in your presence. It is a girl, six years of age. She presents a defect of fœtal development. The anterior abdominal walls have never completely closed along the median line of the body. There is no umbilicus. The recti muscles separate at a point above where the umbilicus should be, and spread out at an angle, until they become attached to the pubes at quite a distance one from the other. The pelvic bones themselves are separated by a wide interval. The anterior wall of the bladder is absent, and the posterior surface of the mucous membrane is presented to view with the ureters discharging upon it. The interior of the bladder is thus exposed to the air. When the patient is at rest the exposed surface is comparatively flat, but when she coughs, vomits, or strains, it rises up and is pushed forward, in shape and size like a billiard ball ; it then bleeds readily, and, as you can imagine, is much irritated by the clothing. There is no urethra, clitoris, or nymphæ. The labia majora lie to the sides by the separated pubic arch. There is a rudimentary vagina, which, as has been noted in other similar cases, lies transversely. The probe enters the vagina an inch and a quarter, and sweeps on either side into a pouch, which is the analogue of the Douglas cul-de-sac in the normal female. The finger introduced into the rectum discovers a small ovoid body, which is probably the rudimentary uterus. The rectum itself appears to be normal.

This is, then, a case of complete exstrophy of the bladder, a disease rare in itself, and especially uncommon in this sex. Of forty recorded cases only seven occurred in females. The inconveniences of the condition are obvious. The child is kept always wet by the trickling urine ; excoriation of the skin follows. The tumor is exposed to a constant friction. The mouths of the ureters being constantly irritated, may become blocked by a phosphatic deposit, which is a source of danger. Yet the condition is not inconsistent with life. This child is likely to grow up. Instances have been recorded in which persons thus afflicted have reached the age of seventy or eighty years.

From separation of the recti, and general weakening of the abdominal walls, we find that this deformity often gives rise in males to double inguinal hernia. In this child the rings are elongated, narrow slits, against which the intestine can be felt on cough, but into which it does not enter. The child is, therefore, thus far free from hernia; moreover, her digestion and other unctions are good, and she may be considered healthy.

Now, on uncovering the parts, you will see that at a point a little below where the umbilicus should be, there is a crescentic line, where the transversalis fascia ends. Below this the tissues are soft, consisting only of the skin and peritonæum over the intestine. Below this is the convex surface of the tumor, presenting a groove with two lateral lobes, and in shape looking not unlike the conventionalized "heart" depicted on playing cards. This is the trigone, and on the edge are the two ureters, which admit, as you see, a fine probe, and are constantly oozing with urine. You see the rudimentary labia, and the absence of the nymphæ. This transverse slit, looking like the meatus, is in reality the ostium vaginæ. Below, the perinæum and raphé are normal. The excoriations about the anus, looking like condylomata, are due simply to the irritation of the urine. The fullness which you see over the separated pubic bones is due chiefly to fat, and in the male often contains a double inguinal hernia. But here, as I have said, the ring is empty. My finger and thumb are on the spines of the pubes, which by measure are nearly three inches apart. Above the bladder the angle formed by the receding recti can be readily seen. The existence of the urachus is denied in these cases. The umbilical cord is attached about at the upper edge of the bladder, and here the umbilical vessels terminate. The deformity is due in these cases to a failure of the anterior layer of the allantois to close. The smallest degree of the same abnormality is seen in the condition known as epispadias. A few years ago there was a man who earned his living by going about the country and exhibiting his extroverted bladder before the various medical classes.

The treatment of this deformity may be mechanical or operative. In the former case a urinal is arranged with a metal cup communicating with a reservoir hanging along the thigh. This is hard to adjust and keep in place, and would be especially so in a child of this age. Operative measures may be divided into two classes, radical and plastic. The former attempts to direct the ureters into the rectum, thus making the lower pouch of the rectum into a bladder, which is to be controlled by the sphincter ani, and which will expell the urine as is necessary, either with or without the fæces. This has often been attempted, and several operations for this purpose have been described. All that 1 have seen, with one exception, have been disastrous. The patient has usually died either from cellulitis or peritonitis. In those that have survived the result has not been successful. The difficulty is that the peritoneum is displaced like all the other viscera, and one cannot tell how low it comes down. Normally it should go no lower than the line of the trigone. In two cases operated upon in London the peritoneum was perforated, and there was intra-peritoneal extravasation of urine. Another difficulty is that even if the operation is primarily successful the ureters may become blocked up by a phosphatic deposit. To perform this operation it is necessary to pass a fine probe into the ureter; the finger is introduced into the rectum. Then two needles are passed from the rectum, and the lower half inch of the ureter is held in the loop of thread. This is then tightened, and the ureter sloughs through into the rectum by an elongated opening.

T. Holmes' method is to slough the whole back wall of the vesical trigone into the rectum by means of a clamp, thus getting a large fistulous opening.

These operations are all open to the objections of being dangerous, and it is uncertain whether the rectum will tolerate the phosphatic deposits, and whether the sphincter will hold well.

The second class of operations are the plastic ones, where the object is to cover in the deformity by flaps of skin. We cannot hope to make a bladder which will hold water, but we cover it with skin in the expectation that the cicatrix will hold back the bladder, and will leave only an exposed point at the base where the ureters will discharge, and to which a urinal can be more accurately fitted.

T. Holmes' mode of operating is to take one square flap from the groin, and reflect it over the bladder, raw side uppermost. A seeond flap is cut obliquely from the other groin and labium or scrotum, and turned or slid over the first, raw surface downwards. A subsequent operation is required to unite the upper edges of these flaps in the wall of the abdomen, above the bladder.

John Wood's operation consists in raising an apron of skin from above the bladder, and turning it down so that the raw surface comes uppermost. Over this are brought two smaller lateral flaps with their raw surfaces downwards; these are joined along the median line, and all are stiched into the margins of the sound skin around the bladder.

Bigelow has grafted the skin directly upon the bladder by dissecting off the mucous membrane above the trigone, raising two flaps at the side, and applying them with the raw surface downward to adhere directly to the bladder, thus occluding the upper three fourths of the viscus.

The operation which I shall do is that of Wood. The assential point is to have no tension, and thus to avoid sloughs. Cleanliness is also especially important. I take the flap from above, about to where the umbilicus should be. The side flaps I make from the groins rather than from the sides of the abdomen, as Wood does, as by his method almost the whole belly is flayed. You will observe that the side flaps taken from the groin infringe upon the erectile tissue about the vulva, and thus give rise to considerable hæmorrhage.

[After this had ceased the flaps were brought into excellent apposition, and stitched with wire and catgut. The protusion of the bladder was entirely closed in except a small opening below. The skin of the belly was drawn together with two button sutures, and the raw surface over the abdomen two thirds closed. The wound was dressed with lint and carbolized oil, one to forty parts.]

